# Renoprotective Effect of a Dipeptidyl Peptidase-4 Inhibitor on Aging Mice

**DOI:** 10.14336/AD.2019.0620

**Published:** 2019-06-20

**Authors:** Tae H Ban, Eun N Kim, Min Y Kim, Ji H Lim, Jong H Lee, Hyung D Kim, Hye E Yoon, Cheol W Park, Bum S Choi

**Affiliations:** Division of Nephrology, Department of Internal Medicine, College of Medicine, The Catholic University of Korea, Seoul, Republic of Korea

**Keywords:** aging kidney, aging, renin-angiotensin system, dipeptidyl peptidase 4, renoprotective effect

## Abstract

Dipeptidyl peptidase 4 (DPP-4) inhibitors exert pleiotropic effects beyond glycemic control. We investigated the renoprotective effects of DPP-4 inhibitors on aging mice mediated by the renin-angiotensin system (RAS). C57BL/6 mice were divided into three groups: the two-month-old mice (YM group), the eighteen-month-old mice (AM group) and the eighteen-month-old, linagliptin-treated mice (AM + LIN group). Renal function was improved, based on serum creatinine and cystatin-C levels (p < 0.05 compared with the AM group for both parameters). Fibrotic areas and the levels of proteins related to fibrosis improved in the AM + LIN group (p < 0.001 compared with the AM group for all parameters). In the AM + LIN group, the DPP-4-positive area and activity and expressions of DPP-4 were decreased (p < 0.05 compared with the AM group for all parameters). The levels of proteins related to the RAS, including prorenin receptor, angiotensin-converting enzyme, angiotensin II and angiotensin 1 receptor, were decreased in the AM + LIN group (p < 0.05, p < 0.01, p < 0.05, and p < 0.01 compared with the AM group, respectively). NADPH oxidase 2 and NADPH oxidase 4 levels decreased in the AM + LIN group (p < 0.001 compared with the AM group for both proteins), whereas the levels of endothelial nitric oxide synthase (eNOS) phosphorylated at serine^1177^ and superoxide dismutase 1 were increased (p < 0.01 compared with the AM group for both proteins). DPP-4 inhibitors may exert renoprotective effects via prorenin receptor/angiotensin-converting enzyme/angiotensin II/angiotensin 1 receptor axis.

The elderly proportion of the population is growing as life expectancy increases in many countries (1-4). In the renal area, renal function and structure deteriorate due to multifactorial and complex processes, including sarcopenia, body weight loss, a decrease in cell number, oxidative stress, chronic inflammation, the renin-angiotensin system and hypertension in the elderly (5-9). In addition to those processes, a decrease in the naturally estimated glomerular filtration rate (eGFR) inevitably causes a greater proportion of the senior population to develop chronic kidney disease (CKD), with an eGFR less than 60 ml/min/1.73 m^2^ (6-8,10,11). Compared to elderly patients with normal renal function, patients with decreased eGFR due to aging presented poor renal and cardiovascular outcomes similar to those of patients with diseases such as diabetes mellitus (DM) and hypertension (HTN), even though the decline in renal function was not disease-related (12). Therefore, the aforementioned pathways were researched to identify possible targets for preventing aging-related processes (6-9). To date, no treatments have had noticeable effects on preserving renal function, except for angiotensin-converting enzyme (ACE) inhibitor and angiotensin II receptor blocker (ARB) (5,13).

Dipeptidyl peptidase 4 (DPP-4) inhibitors are widely used as therapeutic agents for diabetes mellitus, reducing serum glucose levels by inhibiting glucagon--like peptide--1(GLP-1) and gastric inhibitory polypeptide (GIP) degradation (14,15). DPP-4 inhibitors are relatively less restrictive in patients with advanced CKD undergoing dialysis. Recently, growing evidence has indicated beneficial effects of DPP-4 inhibitors in addition to lowering glucose levels (16). For example, past studies have observed renoprotective effects in animal models, including unilateral ureteral obstruction, remnant kidney, ischemia-reperfusion renal injury and drug-induced renal dysfunction (17-21). In addition, positive effects were also proven in cardiovascular disease (16,22,23). The various effects are possible because DPP-4 is expressed in multiple organs, including the kidney, heart, lung, digestive system, and central and peripheral nervous system (24). The kidney is a major organ that expresses DPP-4 in renal blood vessels, glomerular cells, and tubular cells (25). Therefore, the beneficial effects of DPP-4 inhibitors may be independent of the hypoglycemic effect. We hypothesized that the renoprotective effects may be mediated by the renin-angiotensin system (RAS). Therefore, we investigated how DPP-4 inhibitors affect the parameters associated with RAS in animal models of aging-associated renal injury.

## MATERIALS AND METHODS

### Animal Model

Male C57BL/6 mice were divided into three groups as follows: two-month-old mice (YM group, n = 8), eighteen-month-old mice (AM group, n = 8) and eighteen-month-old, linagliptin-treated mice (AM + LIN group, n = 8). All animal research procedures were performed in accordance with the Laboratory Animals Welfare Act, the Guide for the Care and Use of Laboratory Animals and the Guidelines and Policies for Rodent Experiments provided by the IACUC (Institutional Animal Care and Use Committee) at the School of Medicine, The Catholic University of Korea (Approval number: CUMS-2017-0022-03). All mice were purchased from the Korea Research Institute of Bioscience and Biotechnology (Chungcheongbuk-do, Republic of Korea). Mice were housed in a temperature- and light-controlled environment with a 12-h light-dark cycle and had free access to normal chow food. The YM group and the AM group were administered sterile water, and the AM + LIN group was administered water containing 3 mg/kg linagliptin supplied by Boehringer Ingelheim (Ingelheim, Germany) daily for 6 months. The dosage of linagliptin was 3 mg/kg/day based on previous reports (26,27). The mice were sacrificed at the age of 8 months in the YM group and 24 months in the AM and AM + LIN groups.

### Assessment of Renal Function

Renal function was investigated at the initiation and end of the study. The mice were placed in individual mouse metabolic cages (Tecniplast, Gazzada, Italy) to collect urine and provided with water and food for 24 h. Enzyme-linked immunosorbent assay (ELISA) kits were used to measure albuminuria in 24-h urine (Albuwell M, Exocell, Philadelphia, PA, USA) and creatinine concentration (The Creatinine Companion, Exocell) in urine. Measurement of the serum creatinine level was assessed by i-STAT system cartridges (CHEM8+, Abbott Point of Care Inc., Abbott Park, IL, USA). Creatinine clearance was calculated using the standard formula: (urine creatinine (mg/dL) × urine volume (mL/24 h))/(serum creatinine (mg/dL) × 1440 (min/24 h)). The sandwich enzyme immunoassay technique (CUSABIO Biotech, Wuhan, China) was used to measure the serum cystatin-C concentrations in mice from each group according to the manufacturer’s protocols.

### Histological Analyses of Renal Tissue

Kidney samples were fixed in 10% formalin. The tissues were embedded in low-temperature melting paraffin, and then, 4-μm sections were processed and stained with periodic acid-Schiff (PAS) and Masson’s trichrome. Ten fields per section were assessed. The sections of renal tissue were assessed using a color-image analyzer (TDI Scope Eye, Version 3.5 for Windows, Olympus, Tokyo, Japan), and the data were calculated using ImageJ (Wayne Rasband, US National Institutes of Health). The glomerular volume and mesangial area were measured in PAS-stained sections. The relative mesangial area was expressed as mesangial/glomerular surface area. A finding of tubulointerstitial fibrosis was defined as a matrix-rich expansion of the interstitium with tubular dilatation, tubular atrophy, tubular cast formation and thickening of the tubular basement membrane.

### Immunohistochemistry

Deparaffinized tissue sections were processed for immunohistochemistry as described previously (28) using primary antibodies against transforming growth factor-β (TGF-β, R&D Systems, MN, USA), collagen IV (Abcam, Cambridge, UK), monocyte chemoattractant protein 1 (MCP-1, Abcam) and DPP-4 (Abcam). All sections were assessed using a color-image analyzer (TDI Scope Eye, Version 3.5 for Windows, Olympus), and the data were calculated using ImageJ.

### Western Blot Analysis

Total protein was extracted from the whole renal tissues with a Pro-Prep Protein Extraction Solution (Intron Biotechnology, Gyeonggi-do, Republic of Korea) according to the manufacturer’s instructions. The levels of the pro-inflammatory TGF-β (R&D Systems), connective tissue growth factor (CTGF, Santa Cruz Biotechnology, TX, USA), collagen IV (Abcam), fibronectin (Proteintech Group Inc., IL, USA) and β-actin (Sigma Life Science, MO, USA) were analyzed.

Changes in the levels of DPP-4 and proteins related to the renin-angiotensin system were analyzed using the following antibodies: DPP-4 (Abcam), angiotensin-converting enzyme (ACE, Abcam), angiotensin-converting enzyme II (ACE II, R&D Systems), angiotensin II type 1 receptor (AT1R, Santa Cruz Biotechnology), angiotensin II type 2 receptor (AT2R, Novus Biologicals, CO, USA), prorenin receptor (PRR, Sigma Life Science), and Mas receptor (MasR, Novus Biologicals).

Antibodies against endothelial nitric oxide synthase (eNOS, Cell Signaling Technology Inc., MA, USA) and eNOS phosphorylated (phospho) at Ser^1177^ (phospho eNOS, Cell Signaling Technology Inc.) were assessed to evaluate the change in eNOS transcription related to age-related endothelial dysfunction (29). In addition, the levels of NADPH oxidase 2 (Nox2, BD Biosciences, MD, USA) and NADPH oxidase 4 (Nox4, Santa Cruz Biotechnology), which are in the Nox family of NADPH oxidases, were analyzed to confirm the balance between oxidative stress and antioxidant systems (30). Finally, superoxide dismutase 1 (SOD1, Enzo Life Sciences, NY, USA) and superoxide dismutase 2 (SOD2, Enzo Life Sciences) antibodies were used to confirm the antioxidant effect. All antibodies were measured using western blotting analyses.

### Fluorometric Assay of DPP-4 Activity 

DPP-4 activity in kidney tissue was measured using a fluorometric assay kit (BioVision, CA, USA).

### Enzyme Immunoassay

The concentrations of angiotensin II (Ang II) and angiotensin (1-7) (Ang (1-7)) in mouse kidney tissue homogenates were measured using the sandwich enzyme immunoassay technique (CUSABIO Biotech, Wuhan, China) and competitive enzyme immunoassay, respectively, according to the manufacturer’s protocols.

### Cell Culture and in Vitro Assay

We purchased human renal proximal tubular epithelial cells (HRPTEpiCs) from ScienCell (ScienCell, Carlsbad, CA, USA) and cultured them to investigate the effect of linagliptin on cell damage by aging. HRPTEpiCs were grown in T-75 flasks using epithelial cell medium (ScienCell, Carlsbad, CA, USA) containing supplements in a humidified atmosphere of 95% air and 5% CO_2_ at 37°C. Cells were used between passages 10 and 13. For the experiments, we plated HRPTEpiCs in 6-well plates at a density of 3 x 10^5^cells per well, incubated them for 48 h, then changed the medium to epithelial cell medium supplemented with 100 nM Ang II (Sigma Life Science), and cultured the cells in the presence or absence 100 nM linagliptin for 24 h. The cells were harvested at the end of the treatment for further analysis.

### Senescence-associated β--galactosidase (SA β--gal) Staining

To detect senescent cells, we performed staining using a Senescence β-Galactosidase Staining Kit (Cell Signaling Technology Inc.). Experiments were carried out using cells treated with 100 nM Ang II with or without 100 nM linagliptin in 6-well plates for 24 h. Cells were rinsed with phosphate-buffered saline (PBS) and fixed with fixative solution included in the SA β-gal kit for 15 min at room temperature. After washing the plate twice with PBS, β-galactosidase staining solution was added and incubated at 37°C without CO2 in a dry incubator for 24 h. SA β-gal-positive cells were detected by light microscopy.

### Small Interfering RNA (siRNA) Transfection in HRPTEpiCs

Scrambled siRNAs targeting DPP-4 was purchased from Bioneer (Daejeon, Republic of Korea). We cultured cells in 6-well plates for transfection, and when the cells reached 60% confluence, they were transfected with the siRNAs using Lipofectamine 3000 (Invitrogen, Carlsbad, CA, USA), according to the manufacturer’s instructions.

### Statistical Analysis

Data are expressed as the means ± standard errors (SE). Differences between the groups were examined for statistical significance using ANOVA with Bonferroni correction (SPSS version 24.0, IBM Corp., Armonk, NY, USA)). Statistical significance was assumed at p-value < 0.05.

**Figure 1.**
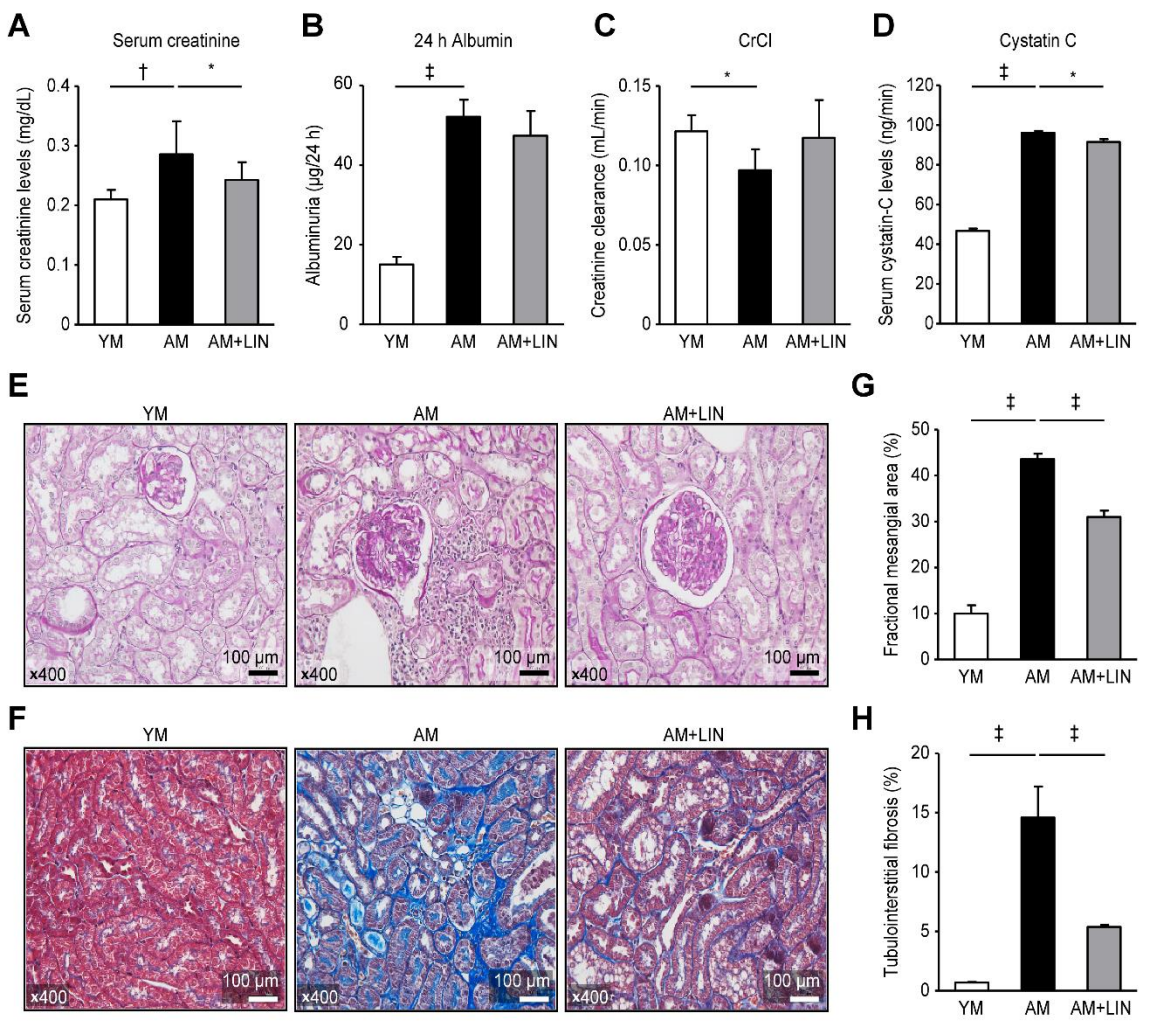
Effects of linagliptin on renal function and aging-related renal injury histology. (A) Serum creatinine levels were increased in the AM group compared to the AM group (p < 0.001), and decreased in the AM + LIN group compared to the AM group (p < 0.05). (B) Albuminuria was increased in the AM group, but it was not different in the AM and AM + LIN groups. (C) Creatinine clearance was decreased in the AM group, but it was similar in the AM and AM + LIN groups. (D) Serum cystatin-C levels were increased in the AM group compared to the AM group (p < 0.001) and decreased in the AM + LIN group compared to the AM group (p < 0.05). (E) The expansion of the mesangial area of PAS-stained kidneys was significantly reduced in the AM + LIN group (original magnification ×400). (F) Decreased tubulointerstitial fibrosis was observed in Masson’s trichrome-stained kidney sections from the AM + LIN group (original magnification ×400). (G) The areas of extracellular matrix in the glomerulus and (H) the areas of tubulointerstitial fibrosis determined using a quantitative assessment were definitely decreased in the AM + LIN group (p < 0.001 for both). (*p < 0.05 and ‡p < 0.001).

## RESULTS

### Effects of Linagliptin on Renal Function in Aging Mice

Renal function was assessed by measuring the serum creatinine level, 24-h urine albumin excretion and creatinine clearance and serum cystatin-C level (Fig. 1). Significantly higher serum creatinine concentrations were observed in the AM group than in the YM group and significantly decreased concentrations were observed in the AM+LIN group compared with the AM group (0.21 ± 0.02, 0.29 ± 0.06, and 0.24 ± 0.03 mg/dL, respectively; p < 0.01 and p < 0.05; Fig. 1A). However, albuminuria was not significantly different between the AM group and the AM+LIN group, despite the higher level of albuminuria in the AM group than in the YM group (15.1 ± 1.94, 52.2 ± 4.32, and 47.4 ± 6.19 mg/day, respectively; p < 0.001; Fig. 1B). Creatinine clearance was lower in the AM group than in the YM group, but it not significantly different between the AM group and the AM+LIN group (0.13 ± 0.01, 0.1 ± 0.01, and 0.12 ± 0.02 mL/min, respectively; p < 0.05; Fig. 1C). On the other hand, a higher serum cystatin-C concentration was observed in the AM group than in the YM group. In the AM + LIN group, the serum cystatin-C concentration was decreased compared to the AM group (46.7 ± 1.24, 96.1 ± 0.92, and 91.6 ± 1.41, respectively; p < 0.001 and p < 0.05; Fig. 1D).

**Figure 2.**
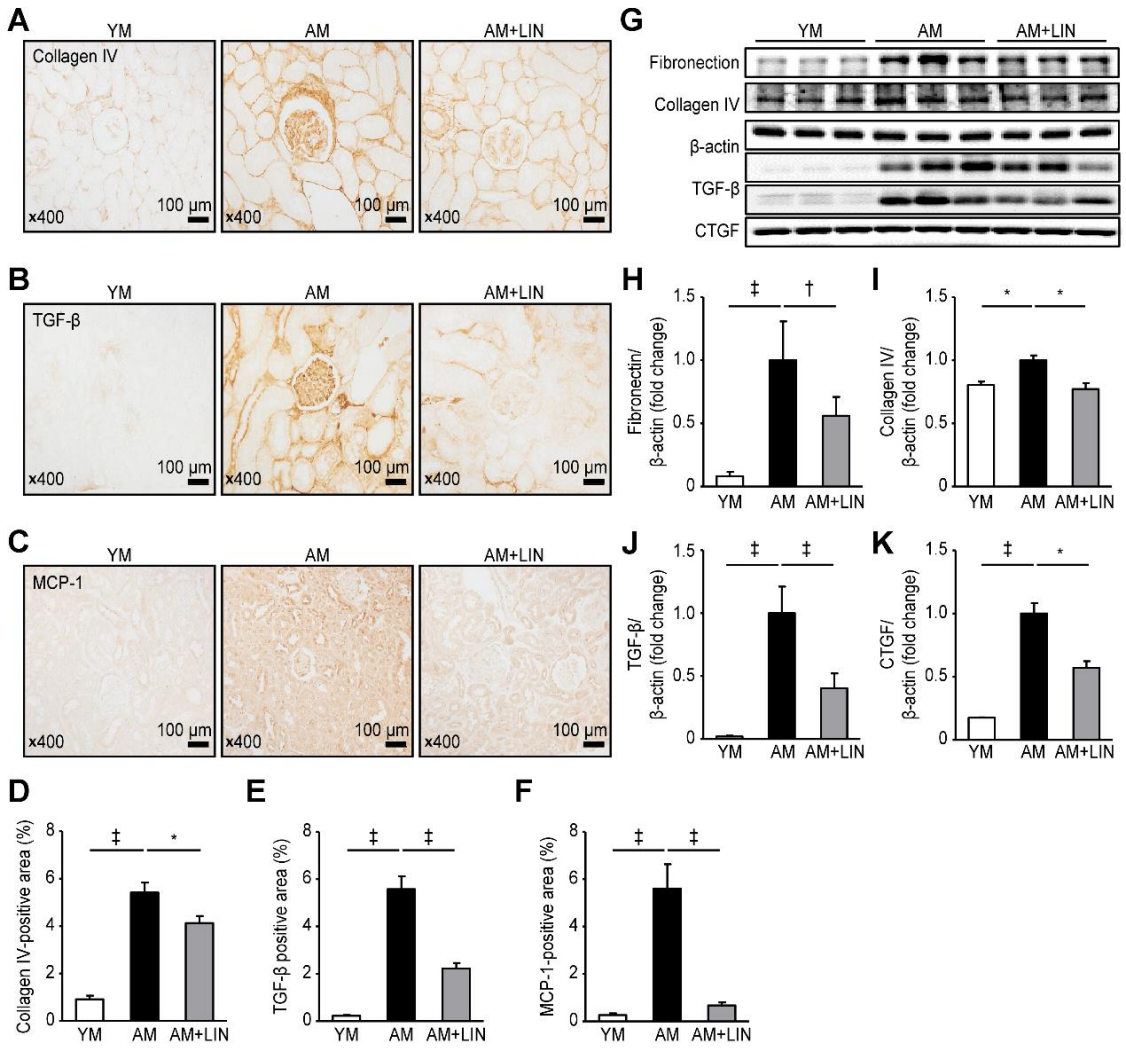
Effects of linagliptin on fibrosis and inflammation in renal tissue. (A) The numbers of collagen IV-, (B) TGF-β- and (C) MCP-1-positive cells were decreased in the AM + LIN group compared to the AM group. (D), (E) and (F) The areas positive for collagen IV, TGF-β and MCP-1 were markedly increased in the AM + LIN group. (G) Levels of markers fibrosis and inflammation were analyzed using western blotting. (H) and (I) Comparison of fibronectin and collagen IV levels between the three groups. (J) and (K) Comparison of TGF-β and CTGF levels between the three groups. (*p < 0.05, †p < 0.01, and ‡p < 0.001).

### Effects of Linagliptin on Renal Histology in Aging Mice

The mesangial area was increased in the AM group compared with the YM group (original magnification ×400; 10.0 ± 1.79% vs. 43.6 ± 1.17%; p < 0.001), but it was decreased in the AM + LIN group compared with the AM group (original magnification ×400; 43.6 ± 1.17% vs. 31.0 ± 1.38%; p < 0.001; Fig. 1E and G). A larger area of tubulointerstitial fibrosis was observed in the AM group than in the YM group (original magnification ×400; 0.70 ± 0.04% vs. 14.6 ± 2.61%; p < 0.001), but it was smaller in the AM + LIN group than in the AM group (original magnification ×400; 14.6 ± 2.61% vs. 5.38 ± 0.17%; p < 0.001; Fig. 1F and H).

**Figure 3.**
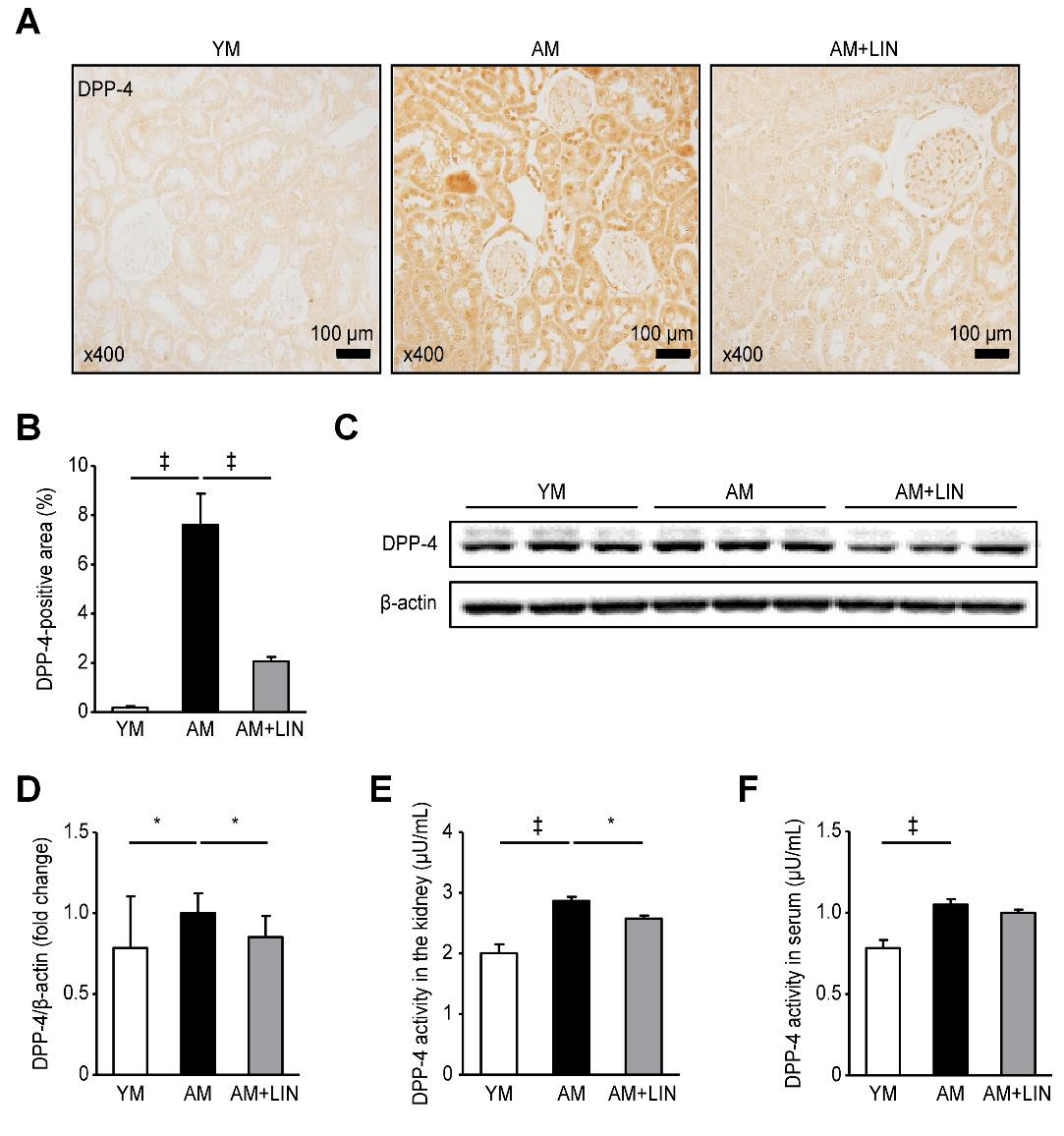
Effects of linagliptin on DPP-4 levels in renal tissues and serum DPP-4 activity. (A) Representative images of immunohistochemical staining for DPP-4 showing positively stained areas in the renal tissue. (B) The DPP-4-positive area was markedly decreased in the AM + LIN group compared with the AM group (‡p < 0.001). (C) Representative western blots showing DPP-4 levels in the renal tissue. (D) Lower levels of the DPP-4 protein were detected in the AM + LIN group than in the AM group (p < 0.05) (E) Lower DPP-4 activity was observed in the AM + LIN group than in the AM group (p < 0.05). (F) The serum DPP-4 activity was increased in the AM group compared with the YM group (p < 0.001), but it was not different between the AM group and the AM + LIN group. (*p < 0.05 and ‡p < 0.001).

### Effects of Linagliptin on Fibrosis and Inflammation in the Renal Tissues of Aging Mice

The fibrotic area was analyzed using collagen IV staining, and the inflammatory cell infiltration was measured by performing TGF-β and MCP-1 immunohistochemistry. A greater fibrotic area expressing collagen IV was observed in the AM group than in the YM group (original magnification ×400; 0.92 ± 0.16% vs. 5.42 ± 0.42%; p < 0.001), but a markedly smaller fibrotic area was observed in the AM + LIN group than in the AM group (original magnification ×400; 5.42 ± 0.42% vs. 4.13 ± 0.30%; p < 0.05; Fig. 2A and D). The infiltration of inflammatory cells, as evidenced by TGF-β staining, was increased in the AM group compared with the YM group (0.23 ± 0.03% vs. 5.58 ± 0.55%; p < 0.001), but it was decreased in the AM + LIN group compared with the AM group (5.58 ± 0.55% vs. 2.23 ± 0.23%; p < 0.001; Fig. 2B and E). The infiltration of inflammatory cells determined using MCP-1 staining was increased in the AM group compared with the YM group (0.27 ± 0.07% vs. 5.60 ± 1.03%; p < 0.001), but it was decreased in the AM + LIN group compared with the AM group (5.60 ± 1.03% vs. 0.67 ± 0.13%; p < 0.001; Fig. 2C and F).

In the renal tissue, differences in fibrosis and inflammation were analyzed using fibronectin, collagen IV, TGF-β and CTGF with western blotting methods. Higher levels of these proteins were observed in the AM group than in the YM group (p < 0.001 for fibronectin; p < 0.05 for collagen IV; and p < 0.001 for TGF-β and CTGF), but the levels were markedly decreased in the AM + LIN group compared with the AM group (p < 0.01 for fibronectin, Fig. 2G and H); p < 0.05 for collagen IV, Fig. 2G and I; p<0.001 for TGF-β, Fig. 2G and J; and p < 0.05 for CTGF, Fig. 2G and K).

**Figure 4.**
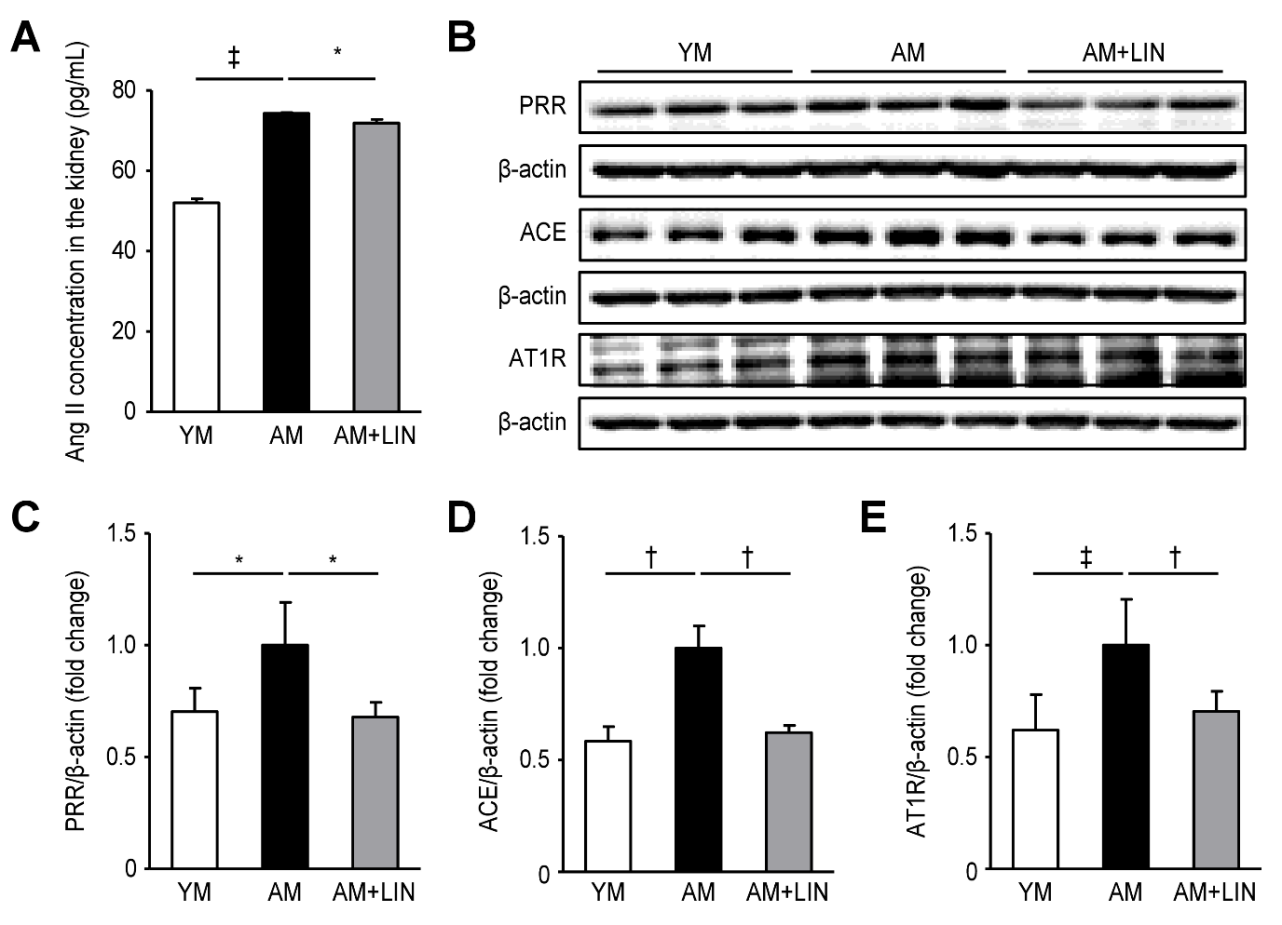
Effects of linagliptin on angiotensin II levels and PRR, ACE and AT1R protein expression in kidneys. (A) The levels of Ang II in the kidneys were decreased in the AM + LIN group compared with the AM group (p < 0.05). (B) Representative western blots of PRR, ACE and AT1R protein expression. (C) Lower levels of the PRR protein were detected in the AM + LIN group than in the AM group (p < 0.05). (D) Lower levels of the ACE protein were observed in the AM + LIN group than in the AM group (p < 0.01). (E) Lower levels of the AT1R protein were observed in the AM + LIN group than in the AM group (p < 0.01). (*p < 0.05, †p < 0.01, and ‡p < 0.001).

### Effect of Linagliptin on DPP-4 Levels in Renal Tissues from Aging Mice

The DPP-4-positive area in renal tissue was analyzed using immunohistochemistry. The size of the DPP-4-positive area was increased in the AM group compared with the YM group (original magnification ×400; 0.19 ± 0.06% vs. 7.62 ± 1.26%; p < 0.001), but it was markedly decreased in the AM + LIN group compared with the AM group (original magnification ×400; 7.62 ± 1.26% vs. 2.07 ± 0.18%; p < 0.001; Fig. 3A and B). The levels of DPP-4 in the renal tissue were evaluated using western blotting. In addition, the activity and concentrations of DPP-4 in the renal tissue and serum were measured using a fluorometric assay. Higher DPP-4 levels and activity were observed in the renal tissue from the AM group than the YM group (p < 0.05 for DPP-4 expression; 2.01 ± 0.15, 2.87 ± 0.07, 2.58 ± 0.05 µU/mL, respectively; p < 0.001 and p < 0.001 for DPP-4 activity, respectively), but significantly lower DPP-4 levels and activity were observed in the AM + LIN group than in the AM group (p < 0.05 for both parameters) (Fig. 3C-E). On the other hand, the activity of DPP-4 in the serum was higher in the AM group than in the YM group (0.78 ± 0.05 vs. 1.05 ± 0.03; p < 0.001; Fig. 3F). However, the activity of DPP-4 in the serum exhibited decreasing trends in the AM + LIN group compared with the AM group, but the difference was not statistically significant.

### Effects of Linagliptin on the Levels of Ang II PRR, ACE, and AT1R in the Kidneys of Aging Mice

The Ang II concentrations in renal tissue homogenates were measured by an enzyme immunoassay. Higher Ang II levels were observed in the AM group than in the YM group, but the levels were significantly decreased in the AM + LIN group compared with the AM group (52.0 ± 1.01, 74.27 ± 0.24, and 71.88 ± 0.95 pg/mL, respectively; p < 0.001 and p < 0.05; Fig. 4A). Subsequently, the expression levels of PRR, ACE and AT1R were assessed by Western blotting. The levels of PRR, ACE and AT1R were increased in the AM group compared with the YM group (p < 0.05 for PRR; p < 0.01 for ACE and AT1R), but the levels of these proteins were significantly decreased in the AM + LIN group compared with the AM group (p < 0.05 for PRR; p < 0.01 for ACE and AT1R; Fig. 4B-E).

**Figure 5.**
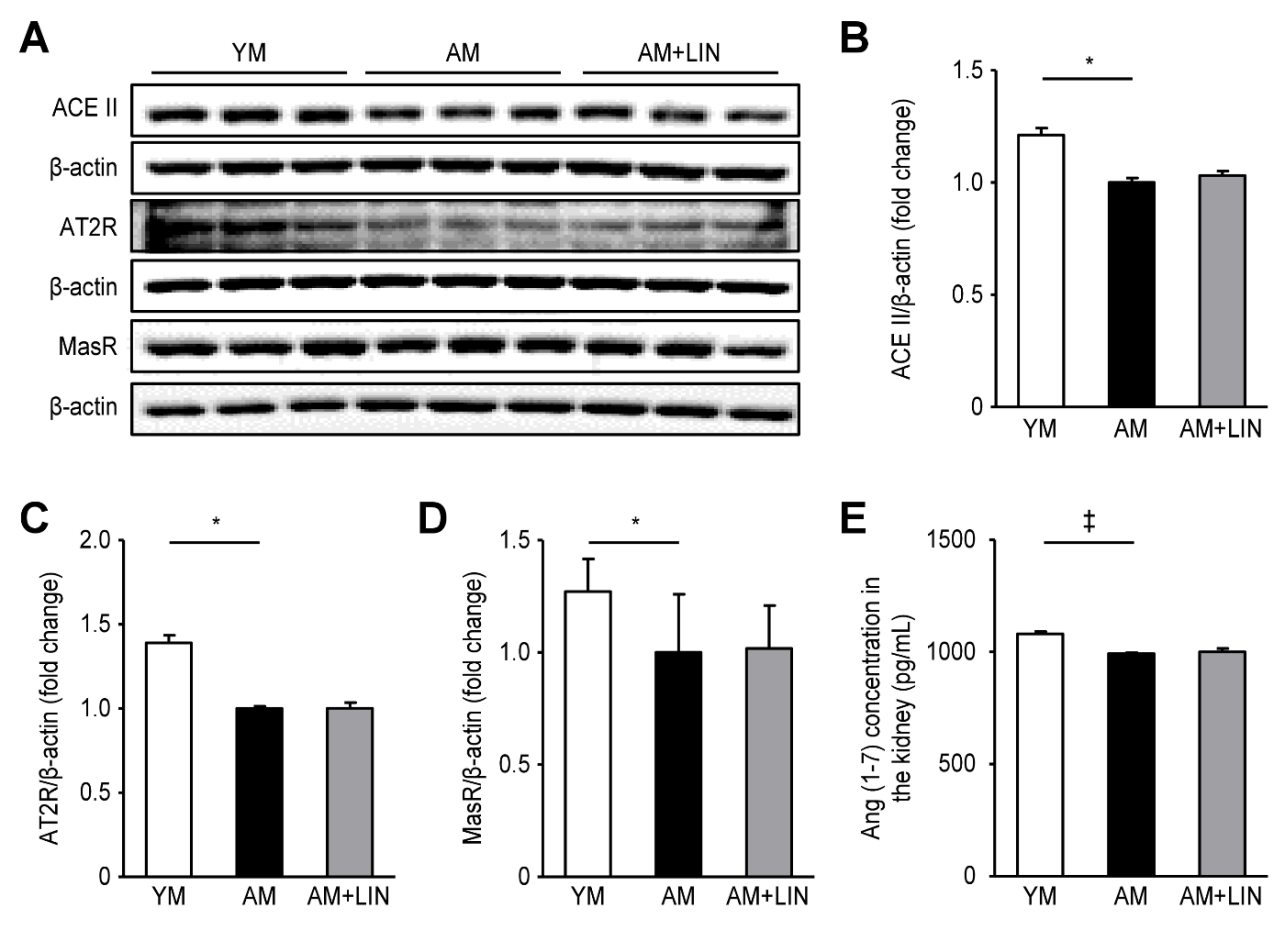
Effects of linagliptin on angiotensin (1-7) concentrations and ACEII, AT2R and MasR protein expression in kidneys. (A) Representative western blots of ACEII, AT2R and MasR protein expression. (B), (C) and (D) Levels of the ACEII, AT2R and MasR proteins were decreased in the AM group compared to the YM group (p < 0.05 for all), but the levels were similar in the AM and AM + LIN groups. (E) Lower levels of Ang (1-7) were detected in the kidneys from the AM group than in the YM group (p < 0.001), but similar levels were observed in the AM and AM+LIN groups. (*p < 0.05 and ‡p < 0.001).

### Effects of Linagliptin on the Levels of Ang (1-7) ACEII, AT2R and MasR in the Kidneys of Aging Mice

The expression levels of ACEII, AT2R and MasR were assessed by western blotting. Lower levels of these proteins were detected in the AM group than in the YM group (p < 0.05 for all), but similar levels were observed in the AM + LIN group and the AM group (Fig. 5A-D). Additionally, the Ang (1-7) concentrations in renal tissue homogenates were measured using an enzyme immunoassay. Lower concentrations were observed in the AM group than in the YM group (p < 0.05), but the levels were not different between the AM + LIN group and the AM group (1080.87 ± 10.27, 993.01 ± 4.10, and 1000.72 ± 14.59 pg/mL, respectively; p < 0.001; Fig. 5E).

### Effect of Linagliptin on the Expression of eNOS, Nox2, and Nox4 in Aging Mice

**Figure 6.**
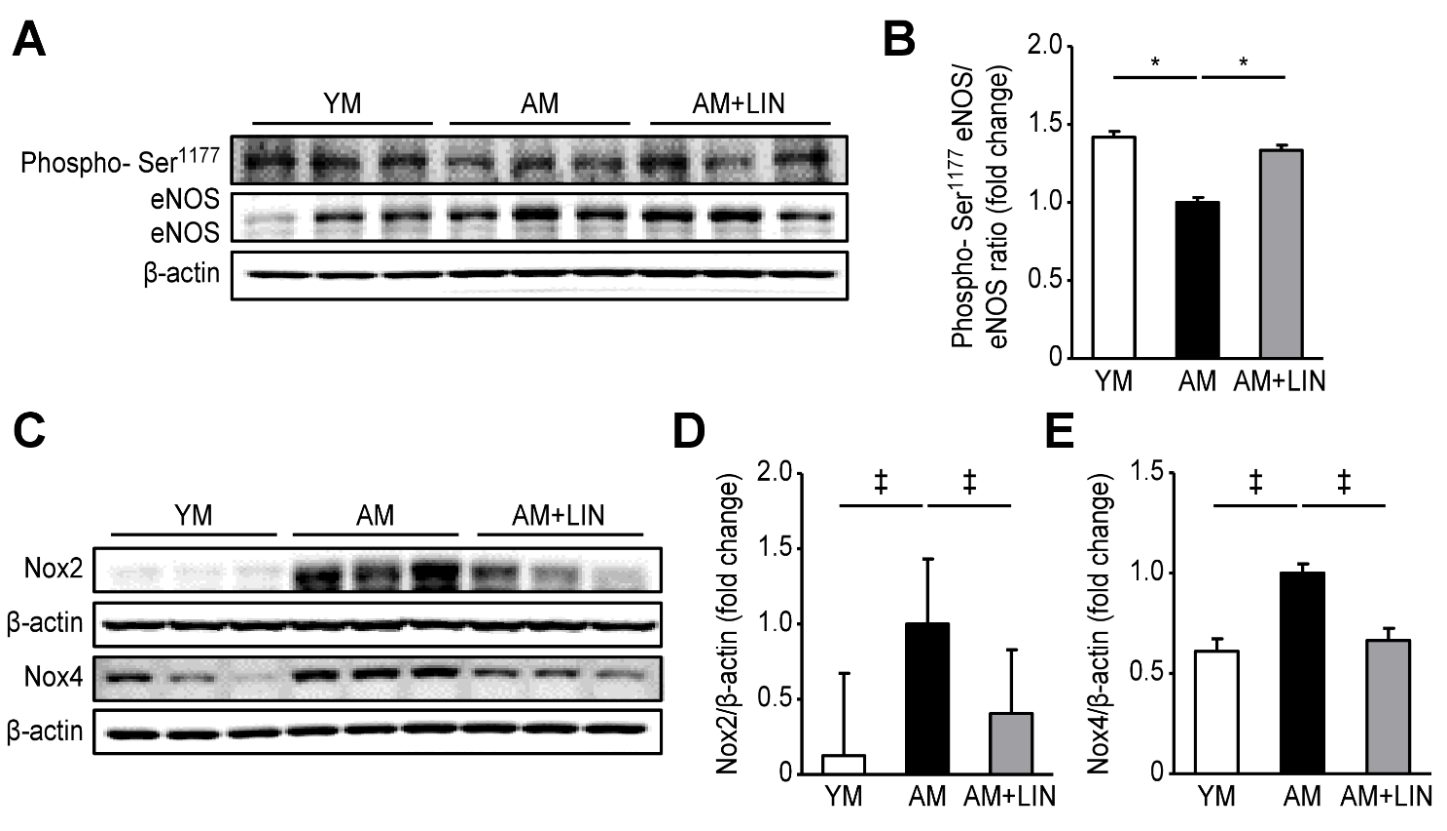
Effects of linagliptin on phospho-Ser1177eNOS/eNOS, NOX2 and NOX4 in renal tissue. (A) Representative western blots of phospho-Ser1177eNOS/eNOS expression. (B) The ratio of phospho-Ser^1177^ eNOS/eNOS was significantly increased in the AM + LIN group (p < 0.05). (C) Representative western blots of Nox2 and Nox4 levels. (D) Nox2 levels were decreased in the AM + LIN group compared with the AM group (p < 0.001). (E) Nox4 levels were significantly decreased in the AM + LIN group (p < 0.001). (*p < 0.05, †p < 0.01, and ‡p < 0.001).

The levels of total eNOS and eNOS phosphorylated at serine1177 (phosphor-Ser^1177^) were analyzed using western blotting to investigate endothelial dysfunction (Fig. 6A and B). The ratio of phospho-Ser^1177^eNOS to eNOS was decreased in the AM group compared with the YM group (p < 0.05), but it was significantly increased in the AM + LIN group compared to the AM group (p < 0.05). A comparison of Nox2 and Nox4, two indicators of reactive oxygen species (ROS), was also performed using western blot analyses (Fig. 6C-E). Higher levels of Nox2 and Nox4 were detected in the AM group than in the YM group (p < 0.001 for both proteins), but lower levels were observed in the AM + LIN group than in the AM group (p < 0.001 for both proteins).

### Effects of Linagliptin on SOD1 and SOD2 Levels in Aging Mice

**Figure 7.**
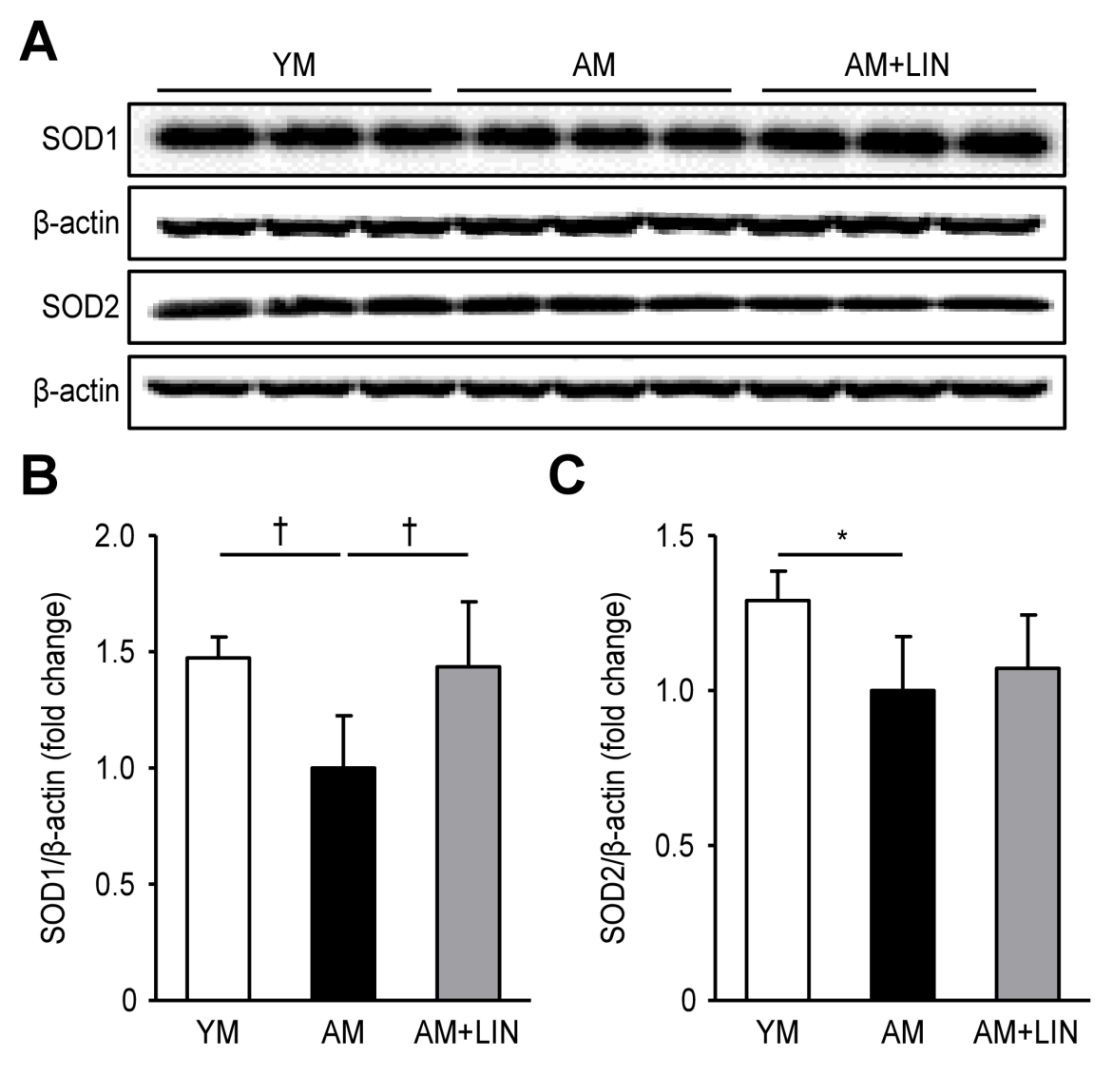
Effects of linagliptin on SOD1 and SOD2 in renal tissue. (A) Representative western blots of SOD1 and SOD2 levels. (B) SOD1 levels were increased in the AM + LIN group compared with the AM group (p < 0.01). (C) SOD2 levels were decreased in the AM group compared to the YM group (p < 0.05), but the levels were not significantly different between the AM group and the AM + LIN group. (*p < 0.05 and †p < 0.01).

SOD1 and SOD2, two indices of antioxidant proteins, were measured via Western blotting analysis. Decreased levels of SOD1 were observed in the AM group compared with the YM group (p < 0.01), but significantly increased levels were observed in the AM + LIN group compared with the AM group (p < 0.01, Fig. 7A and B). Although lower levels of SOD2 were observed in the AM group than in the YM group (p < 0.05), the levels of this protein were not different between the AM group and the AM + LIN group (Fig. 7A and C).

### Effect of Linagliptin on Renal Tubular Cell Injury during Aging in Vitro

**Figure 8.**
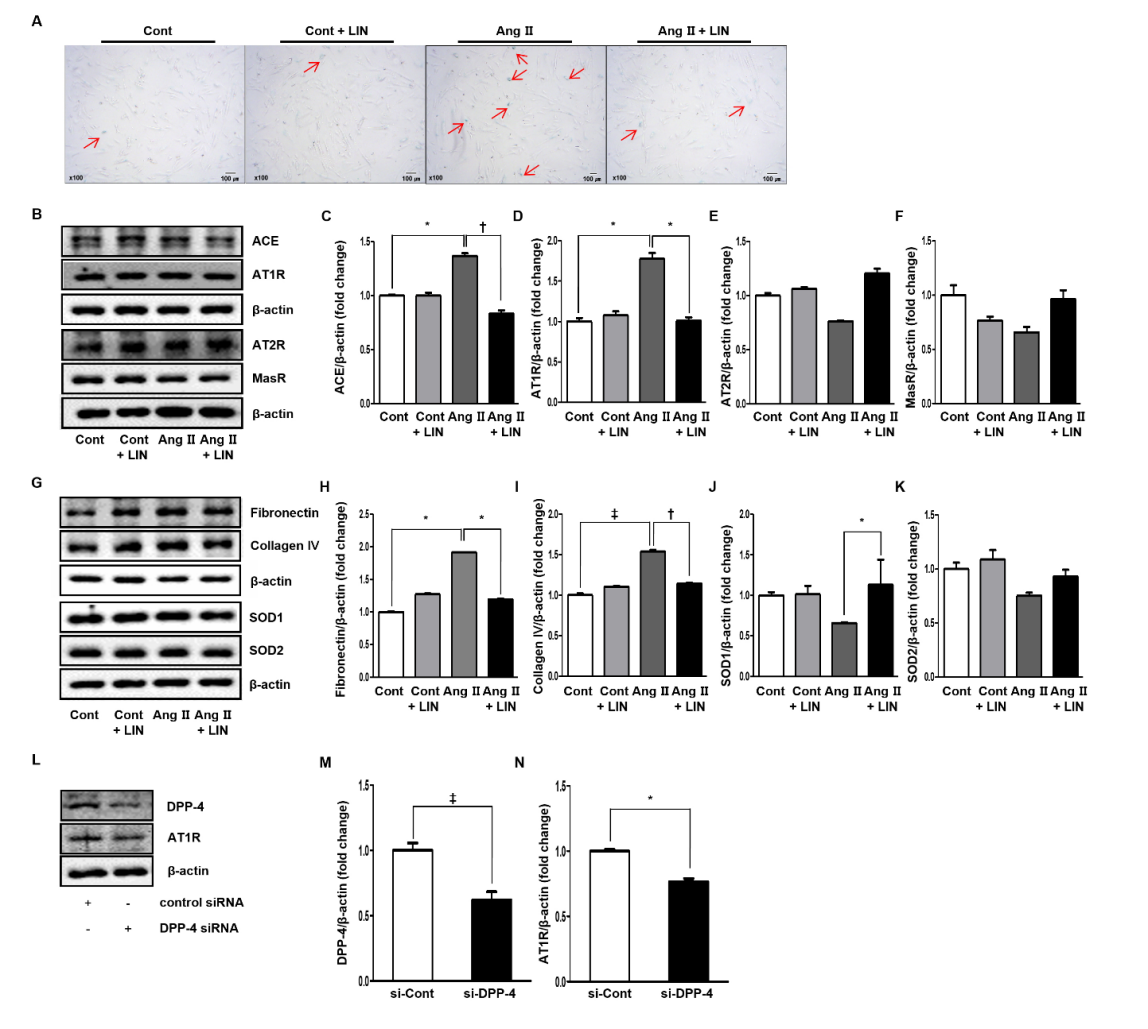
Effects of linagliptin on renal tubular cell injury in HRPTEpiCs. (A) The Ang II-treated group showed an increased number of SA β-gal-positive cells compared to the Cont group. Treatment with LIN decreased the number of SA β-gal-positive cells in the Ang II group, whereas LIN treatment did not affect that in the Cont group. (B) Representative western blots of expression of the renin-angiotensin system in HRPTEpiCs. (C) and (D) Ang II increased the expression of ACE and AT1R compared to the control (p < 0.05 for both), but those effects diminished after adding linagliptin to the Ang II-treated cells (p < 0.01 and p < 0.05, respectively). (E) and (F) The expression levels of AT2R and MasR in the Ang II groups were not different regardless of LIN treatment. (G) Representative western blots of expression of the markers of anti-inflammatory and antioxidant systems in HRPTEpiCs. (H) and (I) The levels of fibronectin and collagen IV were increased in HRPTEpiCs treated with Ang II (p < 0.05 and p < 0.001, respectively), but those effects were stabilized after the administration of linagliptin to Ang II-treated cells (p < 0.05 and p < 0.01, respectively). (J) and (K) The expression levels of SOD1 and SOD2 were not significantly changed in HRPTEpiCs treated with Ang II. The expression of SOD1 improved after adding linagliptin to Ang II-treated cells (p < 0.05), but the expression of SOD2 did not change even after the addition of linagliptin. (L) Representative western blots showing decreased levels of DPP-4 and AT1R in HRPTEpiCs transfected with siRNAs targeting DPP-4. (M) and (N) DPP-4 and AT1R levels were significantly decreased in cells transfected with the siRNA targeting DPP-4 (p < 0.001 and p < 0.05, respectively). (*p < 0.05, †p < 0.01, and ‡p < 0.001).

Ang II-treated cells showed an increased number of SA β-gal-positive cells compared to the control (Cont) cells. Furthermore, treatment with LIN decreased the number of SA β-gal-positive cells in the Ang II group, whereas the LIN treatment did not affect the number in the Cont group (Fig. 8A).

HRPTEpiCs treated with Ang II exhibited increased expression of ACE and AT1R compared to the Cont cells (p < 0.05 for both; Fig. 8B-D), but the expression of AT2R and MasR was not different (Fig. 8B, E and F). After cotreatment with LIN and Ang II, the levels of ACE and AT1R in HRPTEpiCs were restored to normal levels (p < 0.01 and p < 0.05; Fig. 8B-D). However, the levels of AT2R and MasR in the Ang II-treated groups were not different following treatment with or without LIN (Fig. 8B, E and F).

Additionally, antioxidant and anti-inflammatory effects were observed in HRPTEpiCs. The levels of fibronectin and collagen IV were increased in HRPTEpiCs treated with Ang II compared with Cont cells (p < 0.05 and p < 0.001, respectively; Fig. 8G-I), whereas SOD1 and SOD2 levels were decreased in those cells (Fig. 8G, J and K). After cotreatment with LIN and Ang II, the levels of fibronectin and collagen IV in HRPTEpiCs were significantly decreased (p < 0.05 and p < 0.01 Fig. 8G-I). Interestingly, SOD1 levels increased in cells cotreated with LIN and Ang II (p < 0.05; Fig. 8G and J), but SOD2 levels only displayed an increasing trend that was not statistically significant (Fig. 8G and K).

In additional experiments, we transfected cultured HRPTEpiCs with siRNAs to confirm the association of DPP-4 (Fig. 8L-N). DPP-4 and AT1R expression were significantly suppressed by siRNA targeting DPP-4 (p < 0.001 for DPP-4 and p < 0.05 for AT1R; Fig. 8L and M).

## DISCUSSION

This study assessed the beneficial effects of treatment with the DPP-4 inhibitor linagliptin for 6 months on the kidneys of aging mice. Linagliptin ameliorated histological changes, including tubulointerstitial fibrosis and glomerulosclerosis. It nonsignificantly improved trends in creatinine-based renal function and albuminuria. However, cystatin-c-based renal function, a marker that is less affected by age and muscle mass (31), was ameliorated. We investigated the changes in levels of RAS-related proteins in kidneys from aging and young mice and compared them with kidneys from linagliptin-treated aging mice using western blotting. The protective effects were exerted via the selective renin-ACE-Ang II-AT1R axis of RAS. However, ACE2, AT2R and MasR were not significantly changed. Linagliptin also alleviated endothelial dysfunction, ROS formation and oxidative stress changes in aging-related renal injury. These findings reproduced the protective effects of a DPP-4 inhibitor on Ang II-induced renal injury of HRPTEpiCs, consistent with the results observed in aging kidneys. Finally, co-suppression of DPP-4 and AT1R expression in DPP-4 knockdown HRPTEpiCs revealed a correlation between DPP-4 and AT1R.

The kidney is the organ expressing the highest levels of DPP-4 (24). In particular, DPP-4 was expressed in a wider range of preglomerular and mesangial areas, including blood vessels in the kidney (25). Therefore, it can act as a more direct effector of vasoconstriction and dilatation. DPP-4 substrates also exhibit effects such as natriuresis, anti-inflammation, RAS inhibition, and vascular effects in the kidney (32-34). In the present study, linagliptin appeared to markedly suppress DPP-4 expression in the kidney.

Although this study did not examine DPP-4 expression in a model of renal fibrosis, several previous studies reported changes in DPP-4 expression induced by fibrosis around the endothelium, as evidenced by renal histology (35,36). DPP-4 inhibitors also block myofibroblast transformation and the endothelial-to-mesenchymal transition in renal tissue. On the other hand, a study using an endogenous antifibrotic peptide, N-acetyl-seryl-aspartyl-lysyl-proline (AcSDKP), confirmed the reduction in DPP-4 expression around fibrotic areas based on renal histology (37). Therefore, the linagliptin-induced decrease in DPP-4 expression alleviates renal fibrosis in the aging kidney.

The direct association between aging and DPP-4 has not yet been established. Thus, reports confirming changes in DPP-4 in relation to aging are rare. Previous reports revealed that plasma DPP-4 activities and DPP-4 levels in other organs became elevated as aging progressed (38-40). These changes in some organs were attenuated by the DPP-4 inhibitor (20,35,38). Despite the kidney being the primary organ in which DPP-4 is expressed, the results of DPP-4 expression in aging kidneys and responses to DPP-4 inhibitor were deficient. In the present study, linagliptin inhibited DPP-4 activity and expression, consistent with findings from previous studies (18,20,21,35,38). However, serum DPP-4 activity was not inhibited by linagliptin. A few previous studies measuring serum DPP-4 activity reported conflicting results for serum DPP-4 activity following DPP-4 inhibitor administration (39,41). These discrepancies may be explained by the lower levels of DPP-4 in serum compared to other organs, such as the kidney, lung, adrenal gland and liver (24). Therefore, the results for serum DPP-4 activity following treatment with the DPP-4 inhibitor appear to be different from the results obtained from other organs. Consequently, linagliptin-induced inhibition of DPP-4 in aging kidneys is the most important finding of the current study because the wide distribution and physiological effects of DPP-4 on aging kidneys was effectively controlled by a DPP-4 inhibitor. On the other hand, this study did not measure insulin levels, blood glucose levels and blood pressure. Although DPP-4 inhibitors are glucose-lowering agents, DPP-4 inhibitors did not affect insulin levels, blood glucose levels and blood pressure in many kidney disease models used in previous studies, including remnant kidney, cisplatin-induced kidney injury, tacrolimus-induced kidney injury, and ureteral obstruction models (18-21,42). Based on these findings, DPP-4 inhibitors are an appropriate candidate for treating renal injury, independent of their glucose-lowering effects. We suggest that reducing DPP-4 activity and expression through a DPP-4 inhibitor in the kidney inhibits senescence in aging kidneys.

RAS activation is one of the leading causes of the aging process (8,9,43). Generally, increased Ang II concentration plays a key role in CKD progression (13,43). Several studies reported that RAS was also activated in a normal aging mouse model (44-46). Therefore, RAS activation in normal aging-related renal injury can lead to CKD progression, such as deterioration of renal function and histological changes (9). The reason RAS is important in the kidneys is that AT1R and AT2R are widely distributed in the kidney (47). We confirmed that of the two receptors, linagliptin mainly inhibited AT1R. Overall, the results of the two axes showed that linagliptin did not affect the ACE2-Ang (1-7)-MasR-AT2R axis, while the PRR-ACE-ang II-AT1R axis was suppressed. Interestingly, the PRR-ACE-ang II-AT1R axis was important for the development of aging-related renal injury in our previous study (46). Under this rationale, treatment with RAS blockade can delay the deterioration of renal function and has proven potential in elderly patients in some clinical trials (5,48,49). The current study revealed that linagliptin exerted its effect via the PRR-ACE-Ang II-AT1R axis in particular to improve aging-related kidney changes. This result may be a key to understanding the benefit of DPP4 inhibitors in a previous study showing that concurrent use of ACE inhibitors and DPP-4 inhibitors in subjects with metabolic syndrome had a synergistic effect on ACE activity reduction (50). Furthermore, these results suggest that the DPP-4 inhibitor alone may benefit from ACE activity reduction. As a result, the DPP-4 inhibitor may be used as an alternative or with a mixture of RAS inhibitors to achieve renoprotection.

Oxidative stress increased by RAS activation occurred as either cellular ROS accumulation by NADPH oxidase activation or mitochondrial ROS production (16). In our previous study (46), we confirmed that the ratio of phospho-Ser^1177^eNOS to eNOS decreased due to age-related endothelial dysfunction. On the other hand, we observed increased expression of NOX2 and NOX4, members of the NOX family of NADPH oxidases, which regulate the balance between oxidative and antioxidant systems. The association between DPP-4 inhibitors and NADPH oxidase is not well known. Although DPP-4 inhibitor has been reported to have a positive effect on endothelial regulation in some studies (20,38,51), the simultaneous action of RAS and NOX via a DPP-4 inhibitor has not been studied. On the other hand, RAS activation is known to increase NOX expression (52,53). From this perspective, RAS inhibition by ACE inhibitors contributed to NOX stabilization (53). In addition, NOX inhibition or a model of NOX knockdown also inhibited Ang II-induced injury (54). In this regard, our study suggests that DPP-4 inhibitors mitigate endothelial stress and ROS generation through RAS inhibition. Thus, the DPP-4 inhibitor attenuated the effects of aging-related renal injury associated with antioxidant effects via RAS inhibition.

Given these findings, RAS activation is a key mechanism of aging-related renal injury. As the PRR-ACE-Ang II-AT1R axis of the RAS is important for the progression of senescence (43), a DPP-4 inhibitor exhibited the potential to suppress the effects of the PRR-ACE-Ang II-AT1R axis on aging-related renal injury. The positive effects included histologic changes, endothelial dysfunction and oxidative stress through stabilization of RAS by a DPP-4 inhibitor. In this process, the renal tissue was selectively affected by DPP-4. Interestingly, DPP-4 knockdown cells exhibited decreased levels of both DPP-4 and AT1R. Based on these findings, DPP-4 inhibition by linagliptin may suppress AT1R expression in the downstream pathway. Therefore, the DPP-4 inhibitor exerted renoprotective effects on aging kidneys through the selective inhibition of the PRR-ACE-Ang II-AT1R axis.

In conclusion, emerging evidence suggests the protective potential of the pleiotropic effects of DPP-4 inhibitors on many organs. We postulate that the pharmacological efficacy of a DPP-4 inhibitor may attenuate renal injury in aging kidneys by selectively targeting the PRR-ACE-Ang II-AT1R axis.
